# Amelioration of aluminum-induced hepatic and nephrotoxicity by *Premna odorata* extract is mediated by lowering MMP9 and TGF-β gene alterations in Wistar rat

**DOI:** 10.1007/s11356-022-20735-8

**Published:** 2022-05-26

**Authors:** Walaa M. S. Ahmed, Marwa A. Ibrahim, Nermeen A. Helmy, Akram M. ElKashlan, Abeer H. Elmaidomy, Amr R. Zaki

**Affiliations:** 1 Department of Clinical Pathology, Faculty of Veterinary Medicine, Beni-Suef University, Beni-Suef, Egypt; 2 Department of Biochemistry and Molecular Biology, Faculty of Veterinary Medicine, Cairo University, Giza, Egypt; 3 Department of Physiology, Faculty of Veterinary Medicine, Beni-Suef University, Beni-Suef, Egypt; 4 Department of Biochemistry, Faculty of Pharmacy, University of Sadat City, Sadat City, Egypt; 5 Department of Pharmacognosy, Faculty of Pharmacy, Beni-Suef University, Beni-Suef, Egypt; 6 Department of Forensic Medicine and Clinical Toxicology, Faculty of Medicine, Beni-Suef University, Beni-Suef, Egypt

**Keywords:** Aluminum toxicity, Family Lamiaceae, Kidney, Liver, MMP9 gene, *Premna odorata*, TGF-β gene

## Abstract

This study aims to investigate the effect of *Premna odorata* (*P. odorata*) (Lamiaceae) on the hepatic and nephrotoxicity induced by aluminum chloride (AlCl_3_) in rat. Wistar male rats were equally classified into four groups: control, *P. odorata* extract (500 mg/kg B.W.), AlCl_3_ (70 mg/kg B.W.), and *P. odorata* extract plus AlCl_3_ groups. All treatments were given orally for 4 weeks. Serum transaminases and some biochemical parameters, hepatic and renal antioxidant/oxidant biomarker; tumor necrosis factor-α (TNF-α); matrix metalloproteinase (MMP9) and transforming growth factor-β (TGF-β) mRNA expression; histopathological examination of the liver, and kidneys were investigated. The obtained results revealed that AlCl_3_ significantly increased the activities of serum aspartate transaminase, alanine transaminase, and alkaline phosphatase as well as produced a significant increase in total cholesterol, triglyceride, urea, and creatinine concentrations, while there were no changes observed in the total protein, albumin, and globulin concentrations. Also, aluminum administration significantly decreased the reduced glutathione content and increased the catalase activity, malondialdehyde, and TNF-α concentrations in the liver and kidney tissue. Moreover, AlCl_3_ results in congestion, degeneration, and inflammation of the liver and kidney tissue. Co-treatment of *P. odorata* extract with AlCl_3_ alleviated its harmful effects on the previous parameters and reduced the histopathological alterations induced by AlCl_3_. Therefore, *Premna odorata* may have a potent protective effect against oxidative stress induced by Al toxicity through downregulation of MMP9 and TGF-β gene expression.

**Supplementary Information:**

The online version contains supplementary material available at 10.1007/s11356-022-20735-8.

## Introduction

Plant-derived compounds used as drugs are in high demand because of their major therapeutic potential (Jabir et al. 2018). Premna species are widespread throughout tropical and subtropical Australia, Asia, and Africa. They are used in traditional medicine for the treatment of immune-related diseases, skin diseases, inflammation, and stomach disorders. They are also known for their antibacterial and antifungal activities (Mohammad et al. 2019). Previous studies reported hepatoprotective activity of the extract of *Premna tomentosa* leaves against acetaminophen toxicity and dimethyl-nitrosamine (Dianita and Jantan 2017). Additionally, in vitro (using HepG2 cells) and in vivo (using tert-butyl hydroperoxide induced hepatic damage in mice model) evaluation of the hepatoprotective activity of the icetexanes isolated from stem-bark of *Premna tomentosa* was reported by Naidu et al. (2014). Also, *Premna serratifolia* and *Premna corymbosa* showed protective activity against paracetamol and carbon tetrachloride-induced hepatic damage in rats, respectively (Singh et al. 2011; Karthikeyan and Deepa 2010). Ethnomedicines prepared from the leaves of *Premna odorata* are used for the treatment of tuberculosis, phlegm, headache, stomachache, cough, tympanites, beri-beri, heart trouble, dysentery, and abdominal pain and to promote wound healing (Dianita and Jantan 2017). In our previous research, *Premna odorata* was evaluated for its antioxidative capability against neurotoxicity induced by aluminum (Ahmed et al., 2021).

Metallic elements are fundamental environmental components. Their presence is deemed unique in that it is difficult to exclude them from the world entirely with the increasing use of a wide variety of metals in industry and our day-to-day lives (Hassanen et al., 2021). Aluminum (Al) is one of the most abundant metals in the earth’s crust, representing approximately 8% of total mineral components (Al-Olayan et al. 2015). Aluminum toxicity has attracted considerable interest due to its persistence in the environment and bioavailability (Mailloux et al. 2011). It accumulates in all body tissues including the liver, kidneys, spleen, heart, and brain causing damage to the target organs (Gonzalez et al. 2009; Ighodaro et al. 2012). The rat model is widely used to assess the toxicity of Al to prove its toxicity in humans.

The present study was planned to investigate the effectiveness of the *P. odorata* extract to alleviate the hepatotoxicity and nephrotoxicity induced by AlCl_3_ exposure. This target was determined by estimation of some liver and kidney function tests, the concentration of oxidative damage, the activities of some essential antioxidant enzymes, the relative expression concentrations of two inflammatory biomarkers, and the histopathological findings. The liver and kidney were chosen for the current study as they are vital organs documented to be greatly sensitive to metal toxicity.

## Materials and methods

### Chemicals and kits

Aluminum chloride was procured from Sigma-Aldrich (St Louis, MO, USA). The assay kits of aspartate transaminase (AST, COD 11,533), alanine transaminase (ALT, COD 11,531), alkaline phosphatase (ALP, COD 11,591), total cholesterol (TC, COD 21,505), triglycerides (TG, COD 11,528), blood urea nitrogen (BUN, COD 11,517), and creatinine (COD 11,734) were procured from Biosystem SA Barcelona (Spain). Total protein (Ref: 1,001,291) and albumin (Ref, 1,001,022) kits were provided from Spinreact Company (Spain). Analytical kit of catalase (CAT) was purchased from Biodiagnostic company (Giza, Egypt, CA 2517). Reagents for measuring glutathione (GSH) and malondialdehyde (MDA) were purchased from Sigma-Aldrich (St Louis, MO, USA). RNeasy mini kit (Qiagen, Germany) was used.

### Plant extract

*P. odorata* leaves were collected in May 2020 from the Giza Zoo garden, Egypt. *P. odorata* was kindly identified by Dr. Abd El Halim A. Mohammed of the Horticultural Research Institute, Department of Flora and Phytotaxonomy Researches, Dokki, Cairo, Egypt. A voucher specimen (2020-BuPD 45) was deposited at the Department of Pharmacognosy, Faculty of Pharmacy, Beni-Suef University, Egypt.

The air-dried leaves (3 kg) were collected and air-dried in the darkness for 1 month. After drying, the leaves were finely powdered using a CM 290 Cemotec™ laboratory grinder (200–230 V, 50–60 Hz, Foss, Denmark). The finely powdered leaves were extracted by maceration without agitation using 70% ethanol (EtOH), (4 L, 3 X, 4 days each) at room temperature and subsequently concentrated under vacuum at 40 °C using a rotary evaporator (Buchi Rotavapor R-300, Cole–Parmer, Vernon Hills, IL, USA) to afford a 200 g crude extract (Ahmed et al., 2021).

### Metabolic analysis procedure

The crude total extract of *P. odorata* leaves was subjected to metabolomic analysis using analytical techniques of LC-HRESIMS (Elmaidomy et al., 2017). LC-HRESIMS metabolomics analyses were done on an Acquity Ultra Performance Liquid Chromatography system coupled with a Synapt G_2_-HDMS quadrupole time of flight hybrid mass spectrometer (Waters, Milford, MA, USA). Chromatographic separation was carried out on a BEH C_18_ column (2.1 × 100 mm, 1.7 µm particle size; Waters, Milford, MA, USA) with a guard column (2.1 × 5 mm, 1.7 µm particle size) and a linear binary solvent gradient of 0–100% eluent B, over 6 min, at a flow rate of 0.3 mL min^−1^, using 0.1% formic acid in water (*v*/*v*) as solvent A and acetonitrile as solvent B. The injection volume was 2 µL and the column temperature was 40 °C. After chromatographic separation, the metabolites had been detected by mass spectrometry using electrospray ionization (ESI) in the positive mode; the source was operated at 120 °C. The ESI capillary voltage was set to 0.8 kV, the sampling cone voltage was set to 25 V, and nitrogen (at 350 °C, a flow rate (FR) 800 L/h) was used as the desolvation gas and the cone gas (FR 30 L/h). The mass range for TOF–MS was set from mass-to-charge ratio (*m*/*z*) 50–1200. In MZmine 2.12, the raw data were imported by selecting the ProteoWizard converted positive files in the mzML format. Mass ion peaks were detected and followed by a chromatogram builder and a chromatogram deconvolution. The local minimum search algorithm was applied and isotopes were also identified via the isotopic peaks grouper. Missing peaks were detected using the gap-filling peak finder. An adduct search as well as complex search was performed. The processed data set was then subjected to molecular formula prediction and peak identification. The positive and negative ionization mode data sets from each of the respective plant extract were dereplicated against the Dictionary of Natural Products (DNP) database.

### Animals and the experimental design

Forty mature Wistar male rats (weighing 125–150 g) were maintained at a controlled temperature (18–20 °C) and humidity (55% ± 10%) with 12 h light/dark cycles and had access to food and water ad libitum. One week after the adaptation period, the rats were randomly divided to four equal groups of 10 rats in each: control, *P. odorata* extract, AlCl_3_, and *P. odorata* + AlCl_3_ groups. The control group was given distilled water by oral gavage. In the second group, the *P. odorata* extract was orally given at a dose of 500 mg/kg B.W. Aluminum chloride was prepared by dissolving the powder in distilled water and administered orally at a dose of 70 mg/kg B.W. (Kadhem and Enaya 2018) to rats in the third group, while rats in the fourth group were given AlCl_3_ followed by *P. odorata* extract after 10–15 min. The treatments were done once daily for four successive weeks. At the end of the experimental period, each rat was anesthetized with 5% diethyl ether and ACE mixture for 2 min and blood samples were collected from the retro-orbital plexus for separation of adequate serum which preserved at − 80 °C for biochemical estimation. After blood collection, the rats were euthanized by cervical dislocation; liver and kidney were carefully dissected and washed with saline. The organ samples were preserved at − 80 °C until processed for total RNA isolation to determine matrix metalloproteinase (MMP9) and transforming growth factor-β (TGF-β) mRNA expression. Also, samples from liver and kidney were homogenized in 10% w/v phosphate buffer (pH 7.4), centrifuged at 10,000 × *g* for 15 min at 4 °C. The obtained supernatant was used for measuring GSH content, CAT activity, MDA, and tumor necrosis factor-α (TNF-α) concentrations. For the histopathological investigation, samples from the liver and kidney were preserved in 10% formalin.

### Biochemical evaluation

#### Hepatic enzymes

The serum liver enzymes, AST, ALT, and ALP activities were measured according to the method of Friedman and Young (2001).

#### Total protein and albumin

Serum total proteins and albumin concentrations were determined according to the method of Peters (1968) and Doumas and Biggs (1972), respectively. The globulin concentration was determined by subtracting the albumin value from the value of total protein to obtain the albumin-globulin (A/G ratio).

#### Total cholesterol and triglycerides

Serum total cholesterol and triglycerides were assayed using a commercial enzymatic kit according to the manufacturer’s instructions.

#### Kidney function

The concentration of BUN and creatinine were assayed with a commercially available assay kit according to the operating instructions.

### Antioxidant/oxidant biomarkers

#### Reduced glutathione

The GSH content was determined according to the method of Van Doorn et al. (1978). This method is based upon the yellow color that is developed by adding 5,5′-dithio-bis (2-nitrobenzoic acid) (DTNB) reagent to sulfhydryl compounds.

#### Catalase

The decomposition of H_2_O_2_ catalyzed by CAT can be measured at 240 nm according to Aebi (1984).

#### Malondialdehyde

The principle of the assay depends on that MDA, the end product of the polyunsaturated fatty acids that is considered an index to measure the concentration of lipid peroxidation MDA reacts with the thiobarbituric acid in an acidic medium to give a pink color that can be assessed spectrophotometrically at 535 nm (Uchiyama and Mihara 1978).

The protein concentration in the supernatant of the liver and kidney homogenates was measured following the method of Lowry et al. (1951). The protein content was determined using Folin’s reagent. The reduction of the phosphomolybdic–phosphotungstic reagent by the copper-treated protein in an alkaline medium at room temperature resulted in blue color.

### Tumor necrosis factor-α

ELISA kit was used to measure the concentration of pro-inflammatory cytokine TNF-α in liver and kidney homogenate following the manufacturer’s instructions using a test reagent kit (eBioscience Company, North America, USA).

### Quantitative real-time PCR for matrix metalloproteinase and transforming growth factor-β genes

Total RNA was isolated from liver and kidney tissues using RNeasy mini kit (Qiagen) according to the manufacturer’s instructions. First-strand cDNA was generated by reverse transcription of 10 μg RNA samples. The primer set used for MMP9 are forward primer: 5′-GATCCCCAGAGCGTTACTCG-3′; reverse primer: 5′-GTTGTGGAAACTCACACGCC-3′ and those of the TGF-β were forward primer: 5′-ACTCCCGTGGCTTCTAGTG-3′; reverse primer: 5′-GGACTGGCGAGCCTTAGTTT-3′. Real-time PCR was done using a Real-Time PCR System (Applied Biosystems, USA) which was run for 40 cycles of denaturation at 95 °C for 30 s, annealing at 59 °C for both genes for 30 s and extension at 72 °C for 30 s. The GAPDH gene was amplified in the same reaction to serve as the internal control (Ibrahim et al. 2020). Each assay was repeated twice, and the values were used to calculate the gene/GAPDH ratio, with a value of 1.0 used as the control (calibrator) (HelmyAbdou et al. 2019). The normalized expression ratio was calculated using the Mxpro software (Khalaf et al. 2019).

### Histopathological examination

The liver and kidney were dissected and collected carefully from all experimental groups. The specimens were fixed in 10% formalin solution for 3 days, after that they were washed in water, dehydrated in ascending grades of alcohol, then they cleared in xylene. Finally, the specimens were embedded in paraffin to prepare paraffin sections (5 µm) and stained with hematoxylin and eosin according to Bancroft and Gamble (2008).

### Statistical analysis

Statistical analysis of results was performed by one-way analysis of variance (one-way ANOVA), followed by Tukey’s post hoc test using GraphPad Prism software (version 5.0) (San Diego, CA, USA); *p* value less than 0.05 is statistically significant. All the data are expressed as mean ± standard error.

## Results

### Metabolic analysis

Chemical profiling of the secondary metabolites of *P. odorata* leaves, using LC-HRESIMS for dereplication purposes, resulted in the characterization a variety of constituents, including sterols, triterpenes, fatty acids, iridoid, flavones, acylated rhamnopyranoses, and phenyl ethanoids (Table S1, Figure S1).

### Biochemistry

The effect of aluminum administration and *P. odorata* treatment on the liver enzymes, lipid, protein profile, urea, and creatinine concentrations are shown in Table 1. Treatment with *P. odorata* extract alone had no significant effect on the activities of AST, ALT, ALP compared to the control. Administration of AlCl_3_ resulted in significant increases in the activities of these enzymes. The serum AST and ALT activities were returned to normal values in the rats in the AlCl_3_ + *P. odorata* group. However, the ALP activity persisted higher compared to the control and *P. odorata* groups.

**Table 1:** Serum biochemical parameter values in different experimental groups

**Parameters**	**Control**	***P. odorata***	**AlCl**_**3**_	**AlCl**_**3**_** + *P. odorata***
**AST (U/l)**	51.0 ± 1.1	53.1 ± 2.0	70.1 ± 2.5^a,b^	56.8 ± 1.6^c^
**ALT (U/l)**	36.6 ± 2.1	37.7 ± 0.9	51.3 ± 1.3^a,b^	40.3 ± 0.86^c^
**ALP (U/l)**	81.1 ± 1.9	82.6 ± 1.8	117 ± 3.6^a,b^	97 ± 2.5^a,b,c^
**TG (g/dl)**	130.6 ± 4.5	126.8 ± 1.5	180 ± 3.9^a,b^	141.8 ± 4.5^c^
**TC (g/dl)**	89.6 ± 3.5	85.8 ± 3.3	196 ± 10.5^a,b^	125.2 ± 9.0^a,b,c^
**Total protein (g/dl)**	6.00 ± 0.20	5.6 ± 0.27	5.4 ± 0.15	5.7 ± 0.20
**Albumin (g/dl)**	3.4 ± 0.15	3.3 ± 0.07	3.5 ± 0.06	3.5 ± 0.1
**Globulin (g/dl)**	2.6 ± 0.08	2.4 ± 0.20	2.0 ± 0.14	2.2 ± 0.17
**A/G ratio**	1.32 ± 0.06	1.34 ± 0.09	1.70 ± 0.17	1.63 ± 0.13
**Urea (mg/dl)**	31.0 ± 0.80	31.8 ± 1.00	38 ± 0.31^a,b^	33.8 ± 1.00^c^
**Creatinine (mg/dl)**	0.82 ± 0.05	0.88 ± 0.06	1.3 ± 0.14^a,b^	1.1 ± 0.10

The results are expressed as means ± SE (*n* = 5) with dissimilar superscript letters

^a^significantly different from control

^b^significantly different from the *P. odorata* group

^c^significantly different from the AlCl_3_ group (*p* < 0.05 considered to be statistically significant)

Treatment with *P. odorata* extract alone did not affect TC and TG as compared with the control. The total cholesterol and TG concentrations were significantly increased by AlCl_3_ treatment as compared with the control. In the AlCl_3_ + *P. odorata* group, the TG and the TC concentrations were significantly decreased when compared to AlCl_3_ group. However, the TC was still significantly higher than the control and *P. odorata* groups. The mean concentrations of the total protein, albumin, globulin, and A/G ratio did not change by AlCl_3_ exposure as compared to the control.

Concerning the concentration of urea and creatinine, there were no significant difference between the *P. odorata* group and the control group. Aluminum alone was seen to increase the urea and creatinine concentrations compared to the control and *P. odorata* groups. Co-administration of *P. odorata* extract with AlCl_3_ significantly decreased the urea concentration and insignificant decrease in the creatinine value compared to the Al group.

### Biomarkers of oxidative stress and TNF-α

The concentrations of GSH; MDA and TNF-α and CAT activity of the liver and kidney tissues are shown in Table 2. Rats treated with *P. odorata* extract alone do not produce any significant alteration in the CAT activity, GSH, MDA, and TNF-α concentrations when compared to the control group. Administration of AlCl_3_ resulted in significant decrease in GSH content, while the CAT activity; MDA and TNF-α concentrations were significantly increased compared to control and *P. odorata* groups. On the other hand, co-administration of *P. odorata* with AlCl_3_ increased the GSH content; a decrease in CAT activity; MDA and TNF-α concentrations in comparison with AlCl_3_ group. However, the MDA concentration was still higher in AlCl_3_ + *P. odorata* group than those in the control group and *P. odorata* groups.

**Table 2:** Values of antioxidant/oxidant and TNF-α in liver and kidney homogenates in different experimental groups

**Parameters**		**Control**	***P. odorata***	**AlCl**_**3**_	**AlCl**_**3**_** + *P. odorata***
**GSH (µM/mg protein)**	Liver	251.0 ± 8.5	257 ± 7.7	178 ± 2.5^a,b^	245 ± 7.5^c^
	Kidney	374 ± 5.3	381 ± 4.9	196 ± 6.6^a,b^	362 ± 7.6^c^
**CAT (IU/min/mg protein)**	Liver	0.71 ± 0.06	0.72 ± 0.04	0.97 ± 0.05^a,b^	0.77 ± 0.04^c^
	Kidney	0.28 ± 0.01	0.30 ± 0.01	0.38 ± 0.02^a,b^	0.32 ± 0.01^c^
**MDA (nM/mg protein)**	Liver	186 ± 6.6	171 ± 12.2	322 ± 14.4^a,b^	238 ± 7.3^a,b,c^
	Kidney	229 ± 7.4	238 ± 3.2	595 ± 12.7^a,b^	328 ± 4.7^a,b,c^
**TNF-α (ng/100 g tissue)**	Liver	142 ± 9.4	145 ± 10.0	261 ± 12.4^a,b^	183 ± 9.6^c^
	Kidney	133 ± 3.7	132 ± 6.0	255 ± 17.6^a,b^	155 ± 8.2^c^

The results are expressed as means ± SE (*n* = 5) with dissimilar superscript letters

^a^significantly different from control

^b^significantly different from the *P. odorata* group

^c^significantly different from the AlCl_3_ group (*p* < 0.05 considered to be statistically significant)

### mRNA expression of TGF-β and MMP9 genes

**Fig. 1:**
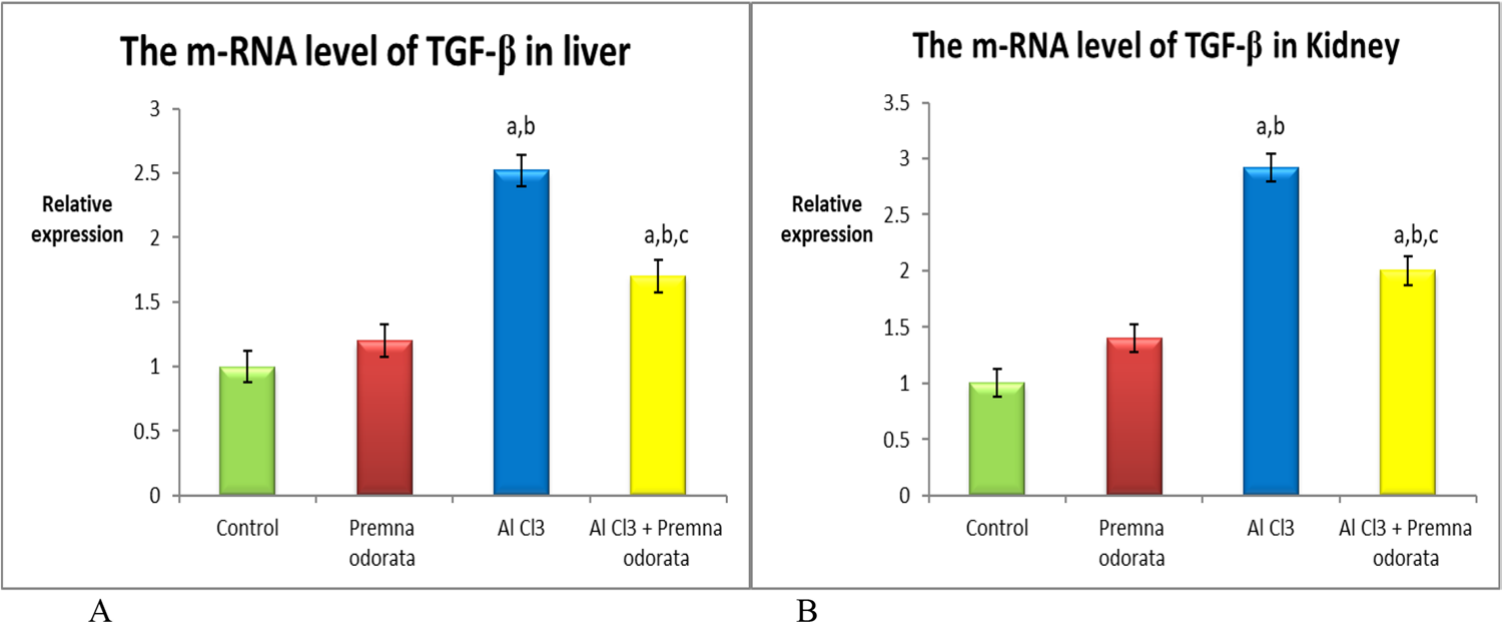
The relative expression level of TGF-β **A** in liver and **B** in kidney of different experimental groups. The dissimilar superscript letters (significantly differing at *p* < 0.05): (a) significantly different from control; (b) significantly different from the *P. odorata* group, and (c) significantly different from the AlCl_3_ group

**Fig. 2:**
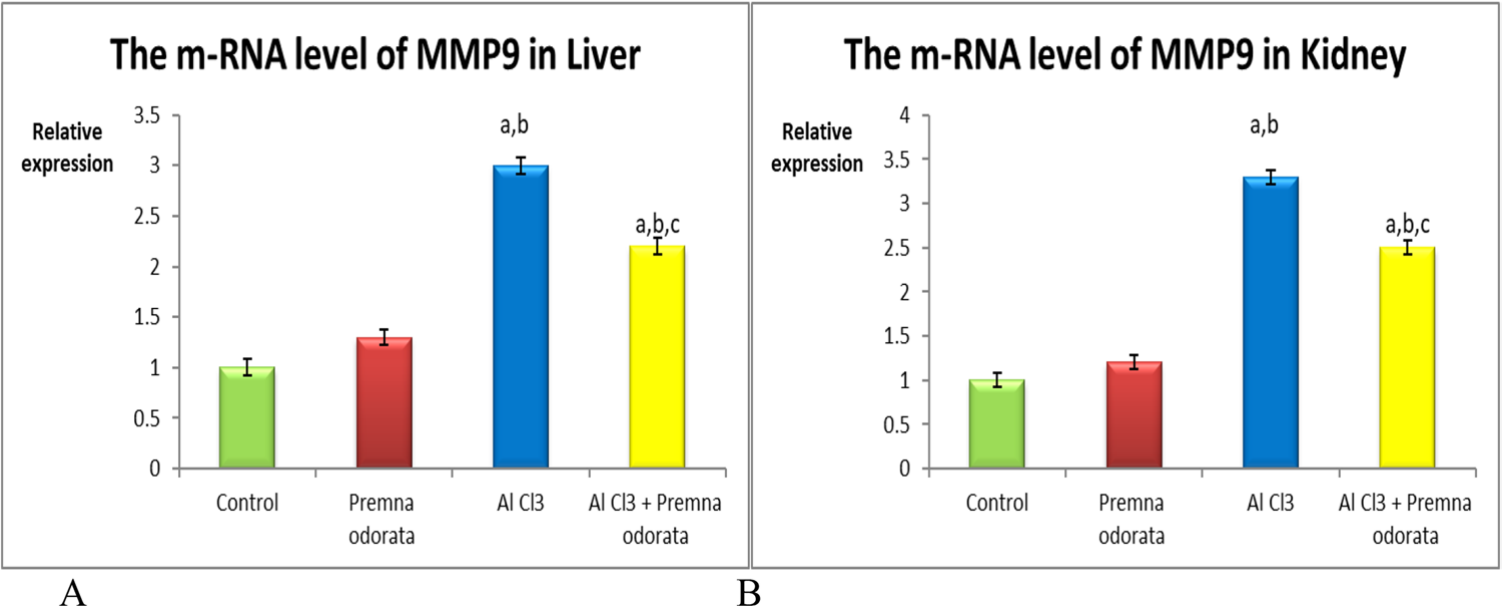
The relative expression level of MMP9 **A** in liver and **B** in kidney of different experimental groups. The dissimilar superscript letters (significantly differing at *p* < 0.05): (a) significantly different from control; (b) significantly different from the *P. odorata* group, and (c) significantly different from the AlCl_3_ group

A significant upregulation in the m-RNA concentration of both TGF-β (Fig. 1) and MMP9 (Fig. 2) genes were detected in the AlCl_3_-treated group relevant to all the experimental groups. Co-administration of *P. odorata* decreased the expression of the studied genes compared to the intoxicated group. These findings were detected in liver and kidney samples.

### Histopathology

**Fig. 3:**
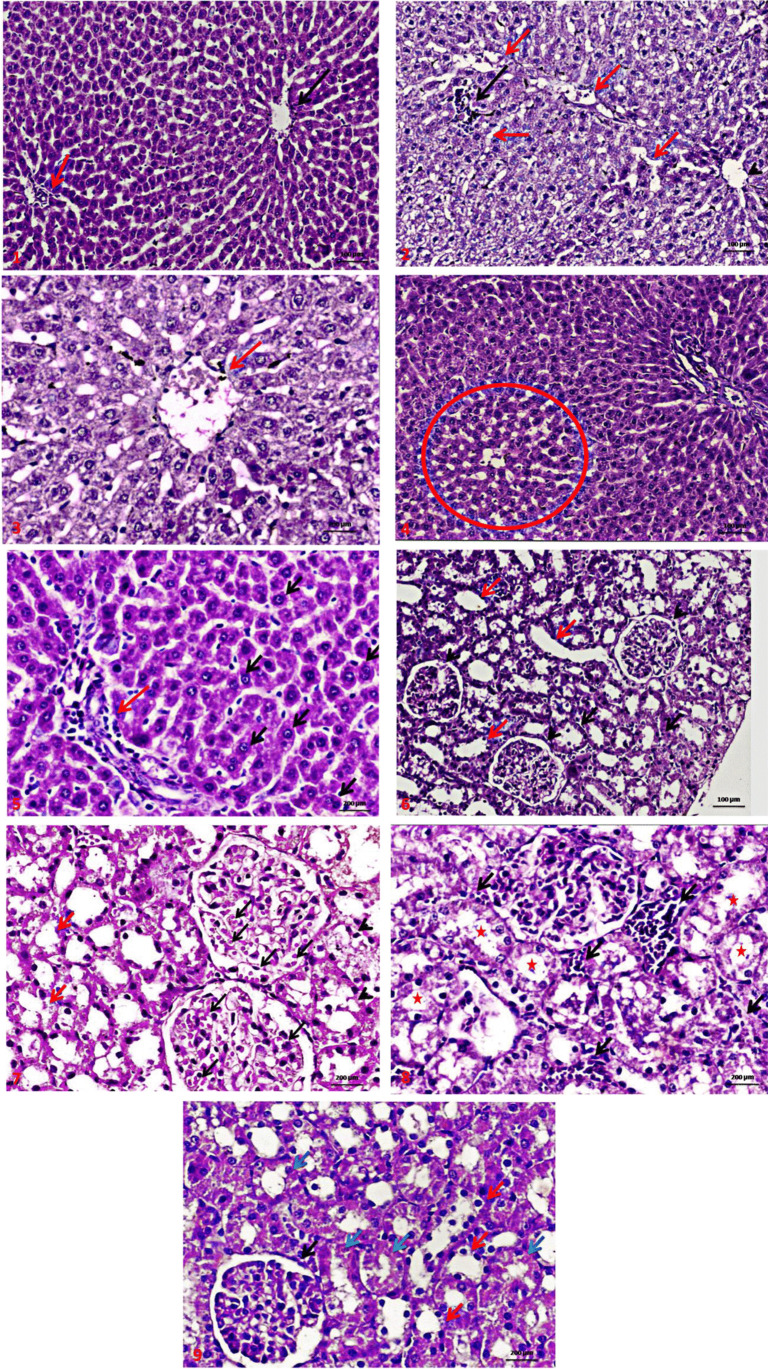
**A** A photomicrograph (H&E × 200) of a section in liver of control rat showing normal liver cell plates formed of polygonal hepatocytes with granular eosinophilic cytoplasm and vesicular nuclei and intervening regular sinusoids and normal central veins (black arrows) with normal portal tact (red arrow). **B** A photomicrograph (H&E × 200) of the liver of AlCl_3_-treated rat showing mild central vein dilatation (arrow head), the liver parenchymal cells revealed diffuse moderate ballooning degeneration of hepatocytes with sinusoidal dilatation (red arrows) with mild lymphocytic parenchymatous aggregations (black arrow). **C** A photomicrograph (H&E × 400) of group the liver of AlCl_3_-treated rat showing moderate central vein congestion (red arrow) with diffuse marked ballooning degeneration of hepatocytes (enlarged distended hepatocytes with clear cytoplasm without nuclear displacement) with moderate sinusoidal dilatation after administration of AlCl_3_. **D** A photomicrograph (H&E × 200) of a section in the liver of AlCl_3_ + *P. odorata* group showing diffuse hepatocellular regeneration with mild central vein and sinusoidal congestion in centrilobular zone (red circle). **E** A photomicrograph (H&E × 400) of a section in the liver of AlCl_3_ + *P. odorata* treated group showing hepatocellular regeneration (black arrow) with mild congestion and inflammation of portal tact (red arrow). **F** A photomicrograph of renal tissue from control group, showing normal glomeruli formed of capillary tuft surrounded by Bowman’s capsule (arrows head), proximal convoluted tubules with narrow lumen lined by high cuboidal cells with homogeneous eosinophilic cytoplasm (black arrows), and distal convoluted tubules with wide lumen lined by low cuboidal cells (red arrows). **G** A photomicrograph (H&E × 400) of the kidney of AlCl_3_-treated group showing marked congestion in the capillary tuft of the glomeruli (black arrow), diffuse mild hydropic degeneration in the epithelial cells lining renal tubules (red arrows) with focal cystic tubular dilatation (arrows head) at corticomedullary portion. **H** A photomicrograph (H&E × 400) of the kidney of AlCl_3_-treated group showing marked interstitial congestion and inflammation (black arrow) diffuse hydropic swelling in the epithelial cells lining renal tubules with focal cystic tubular dilatation (stars) at corticomedullary portion. **I** A photomicrograph (H&E × 400) of *P. odorata* + AlCl_3_ group, a section in the kidney showing minimal congestion in the capillary tuft of the glomeruli (black arrow) with focal regeneration of epithelial cells lining renal tubules (red arrow) with residual degenerative changes in some renal tubules (blue arrow)

The liver histopathological picture is illustrated in Fig. 3. The liver of control rats showed normal histological structure (Fig. 3A). Liver of AlCl_3_-treated rats showed central vein dilatation and congestion, diffuse ballooning degeneration of hepatocytes with sinusoidal dilatation (Fig. 3B and C). In the AlCl_3_ + *P. odorata* group, the liver showed diffuse hepatocellular regeneration with central vein and sinusoidal congestion in the centrilobular zone with some congestion and inflammation of portal tact (Fig. 3D and E). Administration of AlCl_3_ resulted in marked congestion in the capillary tuft of the glomeruli, diffuse hydropic degeneration in the epithelial cells with focal cystic tubular dilatation (Fig. 3G and H). Co-treatment with AlCl_3_ + *P. odorata* alleviated the effect AlCl_3_ as there was a minimal congestion in the capillary tuft of the glomeruli with focal regeneration of epithelial cells (Fig. 3I).

## Discussion

The liver is considered the main metabolic organ of the body. This makes the liver highly susceptible to harmful metabolites that generated during the metabolism process. In the current study, aluminum-induced hepatotoxicity was demonstrated by an increase in serum AST, ALT, and ALP activities. Serum transaminases are indicative for liver injury, as they are escape from the injured hepatic cells to the plasma following hepatocellular degeneration or injury with alteration in the permeability of liver membrane. This was further confirmed by hepatic histopathological changes including congestion and diffuse degeneration of hepatocytes with sinusoidal dilatation. These results are in agreement with previous studies (Balgoon 2019; Galal et al. 2019; Al-Kahtani et al. 2020). Shati and Alamri (2010) previously suggested that the increase in the serum ALT and AST activities might be due to the leakage of these enzymes from the liver cells into the circulation and/or disturbance in these enzymes biosynthesis and liver dysfunction, with an alteration in the permeability of the hepatocyte membranes. Alkaline phosphatase is a membrane-bound enzyme and it is considered a sensitive biomarker of hepatic injury and because its activity dependent on energy metabolism. A decrease in its activity may indicate impaired cellular energy processing (Al-Hashem 2009).

Administration of *P. odorata* extract simultaneously with AlCl_3_ was able to prevent the upsurge in ALT, AST, and ALP activities. The leaves of *P. odorata* exhibited a wide range of pharmacological activities as they contain bioactive and medicinal compounds which provide a scientific evidence of the medicinal benefits of the *P. odorata* (Pinzon et al., 2011). Also, leaves gave high percent of crude extract comparing with other parts (Elmaidomy, et al., 2019). Also, the yield of extraction depends on the solvent used for extraction. Ethanol has been described as good solvent for the extraction of polyphenol; also it is safe among other solvents for human consumption (Do et al., 2014). Treatment with *P. odorata* extract may inhibit peroxidation of membrane lipids and maintain cell membrane integrity by neutralizing free radicals, thus prevented the leakage of hepatic enzymes (Shati and Alamri 2010).

The elevated concentrations of total cholesterol and triglycerides in AlCl_3_-treated rats may be attributed to the disturbance in lipid metabolism due to Al exposure (Newairy et al. 2009; Shati and Alamri 2010 and John et al. 2015). That is consistent with Al Eisa and Al Nahari (2016) who attributed the increase in the serum cholesterol concentration that was seen in rats given AlCl_3_ to hepatic dysfunction and the elevated serum TG concentration to the hypoactivity of lipoprotein lipase which is responsible for triglycerides degradation. Flavonoids, polyphenols, and phenolic acids in *P. odorata* that possess antioxidant activity might reduce the serum TC and TG concentrations (Aniss et al. 2014). Patel and Patel (2012) reported that the extract of root bark of *Premna integrifolia* significantly decreased serum total cholesterol and triglyceride in rats. Subramani et al. (2017) attributed the anti-atherosclerotic activity of hydroalcoholic extract of root bark of *Premna integrifolia* to its flavonoids and phenols contents.

In the present study, no change was observed in protein profile in AlCl_3_-treated rats. Conversely, a significant decline in the concentrations of total protein and albumin was reported by Newairy et al. (2009) and Al Eisa and Al Nahari (2016) in rats treated with AlCl_3_.

Urine is the primary route for Al elimination (about 95%), while biliary route accounts for < 2% of total Al elimination (Yokel and McNamara 2001). Urea, a nitrogenous end product of amino acids, which is filtered by the glomerulus, reabsorbed by the renal tubules and excreted in urine. Creatinine, a nitrogenous end product of muscle creatine metabolism, is a specific indicator of glomerular function. In the present study, elevation of urea and creatinine concentrations in Al-exposed rats revealed significant renal damage and metabolic disturbances (Yu et al. 2017).

The present findings were in agreement with the results of other studies (Belaïd-Nouira et al. 2013; Al-Kahtani and Morsy 2019 and Balgoon 2019) which detected that Al induces renal damage. In the current study, the increased concentration of urea and creatinine is substantiated by the altered histological feature in the kidney of rats treated with AlCl_3_. Co-administration of *P. odorata* extract improved the biochemical and histological alterations induced by AlCl_3_ in liver and kidney, which could be related to the antioxidant activity. The *P. odorata* contains iridoid glycosides that have been noted to possess significant biological activities such as neuroprotective, anti-diabetic, and hepatocurative activities (Hang et al. 2008).

Reactive oxygen species (ROS) production can be increased due to toxicity, causing significant damage to cellular structures and thus induce oxidative stress (Elhelaly et al. 2019). The oxidative stress has been linked to a series of pathological conditions including inflammation, damages the macromolecular structures in the cell, and cellular dysfunction. The aluminum toxicity is attributed to the increase of ROS generation giving rise to oxidative deterioration of cellular proteins, lipids, and DNA, besides induction of changes in the tissue antioxidant enzymes activities and alteration gene expression (Mailloux et al. 2011).

Yu et al. (2017) reported that aluminum mainly accumulates in the liver and kidney during the first 8 weeks from receiving Al. Aluminum might induce oxidative stress in soft tissues like kidney and liver by decreasing the activity of glutathione-synthase enzyme, thus leading to a reduced GSH content (Orihuela et al. 2005 and Gonzalez et al. 2007). Moreover, the decreased concentration of GSH by Al could be due to insufficient supply of NADPH, which is the main factor for the GSH regeneration, by inhibiting NADPH-generating enzymes such as glucose 6-phosphate dehydrogenase (Newairy et al. 2009).

Catalase is a hydrogen peroxide (H_2_O_2_) scavenger that catalyzes the breakdown of H_2_O_2_ to H_2_O and oxygen molecule to protect cells against the toxic effects of H_2_O_2_ (Chance et al. 1979). The elevation of CAT activity in the Al toxic group found in this study could be an indicator for enhanced free radical generation especially H_2_O_2_, in the liver and kidney tissues (Aniss et al. 2014) and appears to be a response towards increased ROS generation for neutralizing its impact (Kumari et al. 2014). In line with the current results, a study by Al-Amin et al. (2018) demonstrated significant increase in the CAT activity in the cortex and hippocampus of mice as response to the oxidative stress damage induced by lipopolysaccharide. Aniss et al. (2014) reported that CAT activity increased in the heart of mice in adriamycin-induced cardiotoxicity. Also, Szymonik-Lesiuk et al. (2003) reported that catalase mRNA expression and enzyme activity were increased by exposure of hepatocytes to H_2_O_2_.

In the current study, administration of AlCl_3_ increased both hepatic and renal MDA. Aluminum is known to bound with transferrin, an iron carrying protein, thus increasing free intracellular iron (Fe2^+^) and, consequently, membrane lipids become primary targets of oxidative damage leading to lipid peroxidation (Nehru et al. 2007 and Mokrane et al. 2020).

The results of this study are consistent with those of John et al. (2015), Ghorbelet al. (2015), and Khalifa et al. (2020). They observed that Al administration significantly elevated tissue TNF-α. According to Ghorbel et al. (2015), the increase of cytokine expression indicate the disruption in pro-oxidant/antioxidant balance and during hepatocyte damage the activated Kupffer cells release growth factors, cytokines, which have a stimulating effect on proliferation and activation of stellate cells. They also release inflammatory mediators (TNF, IL-1, IL-6, IL-8), which are responsible for infiltration of inflammatory leukocytes. In the current study, administration of *P. odorata* extract to the Al-intoxicated rats significantly decreased the concentrations of TNF-α in liver and kidney compared with those in the AlCl_3_ group. Oral administration of *P. odorata* volatile oils to tuberculosis-infected mice caused a decrease in the elevated serum concentration of TNF-α (Mohammadet al. 2019). Phytochemical studies on *P. odorata* leaves have led to characterization of diosmetin and acacetin as the chemical composition of the *P. odorata* which have anti-inflammatory, anti-microbial, and chemopreventive properties (Tantengco and Jacinto 2015; Pinzon and Uy 2016).

The current results support the conclusion that the AlCl_3_-induced upregulation of TGF-β and MMP9 gene transcription might contribute to disruptive functions in the liver and kidney of the intoxicated rats.

Aluminum chloride was reported to induce cellular damage due to excessive ROS production which causes oxidative stress through disrupting the antioxidant defense mechanism (Rahmani et al. 2020). The important role of oxidative damage in the AlCl_3_ toxicity makes it important to consider antioxidant therapy against the AlCl_3_ toxicity (Ahmad et al. 2018).

Transforming growth factor-β (TGF-β) plays an important role in the control of inflammation as an immune suppressive factor (Han et al. 2012). It may negatively regulate immune cell response by activating regulatory T cells (Tregs) and by inhibiting immune cells proliferation, while it can induce Th17 differentiation and enhance the secretion of proinflammatory cytokine IL-17 (Yoshimura et al. 2010). The cytokine TGF-*β* is the major pro-fibrotic cytokine that is over expressed in generalized inflammation (Samarakoon et al. 2013) and it regulates the recruitment of inflammatory cells and macrophage differentiation.

Upregulation of the MMP expression has been reported in almost all tissue inflammation in human. MMPs are incorporated in the modulation of inflammatory mediators which attract the immune cells to the inflamed tissues (Prabhu and Frangogiannis 2016). Aluminum chloride markedly upregulated the proteins related to cell migration, such as MMP-2 and MMP-9.

Our results suggest that *P. odorata* provides protective effects against AlCl_3_-induced oxidative stress in liver and kidney; thus, it maybe of therapeutic benefit in AlCl_3_-induced hepato-nephrotoxicity.

On the other hand, the administration of antioxidants can reduce the toxic effects of AlCl_3_ (Al Dera 2016). The *P. odorata* ameliorated the AlCl_3_-induced hepato-nephro damage by its potent antioxidant constituents (Elmaidomy et al. 2019) which render the *P. odorata* able to scavenge ROS and modulate the expression of TGF-β and MMP9.

Taken together, these results suggest that the antioxidative and anti-inflammatory properties are possible mechanisms of action of *P. odorata* for the management of pro-oxidants and oxidative stress, which are the most causative factors of Al toxicity (Elmaidomy et al. 2020).

## Conclusion

Based on the current study, *P. odorata* treatment reduced the hepatic enzyme activity; improved lipid profile and kidney function; increased the GSH content; reduced lipid peroxidation (MDA) and proinflammatory marker (TNF-α) as well as downregulated the TGF-β and MMP9 gene expression and minimized the AlCl_3_-induced hepatic and renal histopathological damage. Therefore, *P. odorata* extract exhibited protective effect against Al-induced hepato-nephrotoxicity via reducing the oxidative stress and the inflammatory response, which might attributed to the flavonoids, polyphenols, and phenolic acids in *P. odorata* extract that possess antioxidant activity.

## Supplementary Information

Below is the link to the electronic supplementary material.

Supplementary file1 (DOCX 226 KB)

## Data Availability

All data generated or analyzed during this study are included in this published article.
