# Supplementary material for: Amelioration of aluminum-induced hepatic and nephrotoxicity by Premna odorata extract is mediated by lowering MMP9 and TGF-β gene alterations in Wistar rat
Source: Environ Sci Pollut Res Int. 2022 May 26;29(48):72827–38. doi: 10.1007/s11356-022-20735-8 (PMC9522688; doi:10.1007/s11356-022-20735-8)

**Table S1.** The LC-HRESIMS dereplication results of *Premna odorata* leave crude extract.

| **Nu.** | **Metabolites name** | **Source** | **MF** | **RT(min.)** | ***m/z*** | **Polarity** |
| --- | --- | --- | --- | --- | --- | --- |
| **1** | Orientin | *Muhlenbergiamontana* | C_21_H_20_O_11_ | 6.95 | 449.1117 | [M+H]^+^ |
| **2** | Vitexin | *Premna odorata* | C_21_H_20_O_10_ | 9.71 | 433.1361 | [M+H]^+^ |
| **3** | Premnoside A | *Premna odorata* | C_39_H_44_O_20_ | 11.37 | 833.2746 | [M+H]^+^ |
| **4** | 6- *O*-*α*- *L*-(2''-*O*-*trans*-caffoyl) rhamnopyranosylcatalpol | *Premna odorata* | C_30_H_38_O_17_ | 11.98 | 671.1910 | [M+H]^+^ |
| **5** | 6- *O*-*α- L*-(3''-*O*-*trans*-caffoyl) rhamnopyranosylcatalpol | *Premna odorata* | C_30_H_38_O_17_ | 12.03 | 671.1910 | [M+H]^+^ |
| **6** | 1-*O*-trans-*p-*hydroxycinnamoyl-  2-*O*-trans-caffeoyl-*α*-L-rhamnopyranoside | *Premna odorata* | C_24_H_24_O_10_ | 12.20 | 473.1070 | [M+H]^+^ |
| **7** | Daucosterol | *Premna japonica* | C_35_H_60_O_6_ | 12.33 | 577.1969 | [M+H]^+^ |
| **8** | 6- *O*-*α*- *L*-(4''-*O-trans*-caffoyl) rhamnopyranosylcatalpol | *Premna japonica* | C_30_H_38_O_17_ | 12.57 | 671.1910 | [M+H]^+^ |
| **9** | 6- *O*-*α*- *L*-(2''-*O-trans*-caffoyl, 3''-*O*-*trans-p-*coumaroyl) rhamnopyranosylcatalpol | *Premna odorata* | C_39_H_44_O_19_ | 12.61 | 817.2282 | [M+H]^+^ |
| **10** | Premnoside H | *Premna odorata* | C_39_H_44_O_18_ | 13.00 | 801.2404 | [M+H]^+^ |
| **11** | Premnoside C | *Premna odorata* | C_40_H_46_O_19_ | 13.09 | 831.2411 | [M+H]^+^ |
| **12** | 6- *O*-*α*- *L-*(2''-*O*-*trans*-*p*-methoxycinnamoyl) rhamnopyranosylcatalpol | *Premna japonica* | C_31_H_40_O_16_ | 13.20 | 669.1634 | [M+H]^+^ |
| **13** | 1-*O-trans*-*p*-hydroxycinnamoyl-  3-*O-trans*-caffeoyl-*α*-L-rhamnopyranoside | *Premna odorata* | C_24_H_24_O_10_ | 13.21 | 473.1070 | [M+H]^+^ |
| **14** | Stigmasterol | *Premna odorata* | C_29_H_48_O | 13.33 | 413.2619 | [M+H]^+^ |
| **15** | Acacetin | *Premna odorata* | C_16_H_12_O_5_ | 13.60 | 285.1126 | [M+H]^+^ |
| **16** | Stearic acid | *Premna odorata* | C_18_H_36_O_2_ | 13.60 | 285.1126 | [M+H]^+^ |
| **17** | Premnoside G | *Premna odorata* | C_39_H_44_O_18_ | 13.72 | 801.2404 | [M+H]^+^ |
| **18** | 6- *O*-*α*- *L*-(4''-*O*-*trans*-feruloyl) rhamnopyranosylcatalpol | *Premna japonica* | C_31_H_40_O_17_ | 13.78 | 685.2780 | [M+H]^+^ |
| **19** | 6- *O*-*α*- *L*-(3''-*O-trans*-*p*-methoxycinnamoyl) rhamnopyranosylcatalpol | *Premna japonica* | C_31_H_40_O_16_ | 13.90 | 669.1634 | [M+H]^+^ |
| **20** | 6- *O*-*α*- *L*-(4''-*O-trans*-*p*-methoxycinnamoyl) rhamnopyranosylcatalpol | *Premna odorata* | C_31_H_40_O_16_ | 14.10 | 669.1634 | [M+H]^+^ |
| **21** | *β*-Sitosterol | *Premna odorata* | C_29_H_50_O | 14.19 | 414.1849 | [M+H]^+^ |
| **22** | Verbascoside | *Premna odorata* | C_29_H_36_O_15_ | 14.47 | 625.1396 | [M+H]^+^ |
| **23** | Premnoside F | *Premna odorata* | C_41_H_48_O_18_ | 14.54 | 829.2011 | [M+H]^+^ |
| **24** | Catalpinoside | *Plantago lanceolata* | C_15_H_22_O_10_ | 14.68 | 363.0380 | [M+H]^+^ |
| **25** | Premnoside E | *Premna odorata* | C_41_H_48_O_18_ | 14.73 | 829.2011 | [M+H]^+^ |
| **26** | Linolenic acid | *Premnamicrophylla* | C_18_H_30_O_2_ | 14.81 | 277.1807 | [M-H]^+^ |
| **27** | 6- *O*-*α*- L-rhamnopyranosylcatalpol | *Gmelina arborea* | C_21_H_32_O_14_ | 14.86 | 509.1473 | [M+H]^+^ |
| **28** | Diosmetin | *Premna odorata* | C_16_H_12_O_6_ | 15.09 | 301.2947 | [M+H]^+^ |
| **29** | Premnaodoroside A | *Premna odorata* | C_42_H_66_O_20_ | 15.94 | 891.3561 | [M+H]^+^ |
| **30** | Premnaodoroside B | *Premna odorata* | C_42_H_66_O_19_ | 16.06 | 875.2437 | [M+H]^+^ |
| **31** | Premnaodoroside C | *Premna odorata* | C_42_H_64_O_19_ | 16.12 | 873.3192 | [M+H]^+^ |
| **32** | Premnoside D | *Premna odorata* | C_40_H_46_O_20_ | 16.21 | 847.2782 | [M+H]^+^ |
| **33** | 6- *O-α*- L-(2''-*O-trans*-*p*-coumaroyl) rhamnopyranosylcatalpol | *Verbascum saccatum* | C_30_H_38_O_16_ | 16.26 | 655.2176 | [M+H]^+^ |
| **34** | Premnaodoroside D | *Premnasubscandens* | C_42_H_64_O_20_ | 17.74 | 889.2297 | [M+H]^+^ |
| **35** | Amyrin | *Premna odorata* | C_30_H_50_O | 18.28 | 465.2018 | [M+K]^+^ |
| **36** | Citrostadinol | *Schizandra chinensis* | C_30_H_50_O | 18.33 | 465.1978 | [M+K]^+^ |
| **37** | Luteolin | *Premna odorata* | C_15_H_10_O_6_ | 18.66 | 309.2349 | [M+Na]^+^ |
| **38** | Apigenin | *Premna odorata* | C_15_H_10_O_5_ | 20.82 | 293.2147 | [M+Na]^+^ |
| **39** | Oleanolic acid | *Lanatana camara* | C_30_H_48_O_3_ | 21.20 | 457.2621 | [M+H]^+^ |
| **40** | premcoryoside | *Premnacorymbosu* | C_45_H_58_O_24_ | 21.52 | 983.4891 | [M+H]^+^ |
| **41** | Arjunolic acid | *Premnamicrophlla* | C_30_H_48_O_5_ | 21.64 | 489.2793 | [M+H}^+^ |
| **42** | Eicosanoic acid | *Ipomea ochraceae* | C_20_H_40_O_2_ | 22.67 | 313.2727 | [M+H]^+^ |

MF: molecular formula, RT: retention time, min: minute.

**Figure S1.** Dereplicated metabolites from LC-HRESIMS analysis of *Premna odorata* leaves total crude extractive.


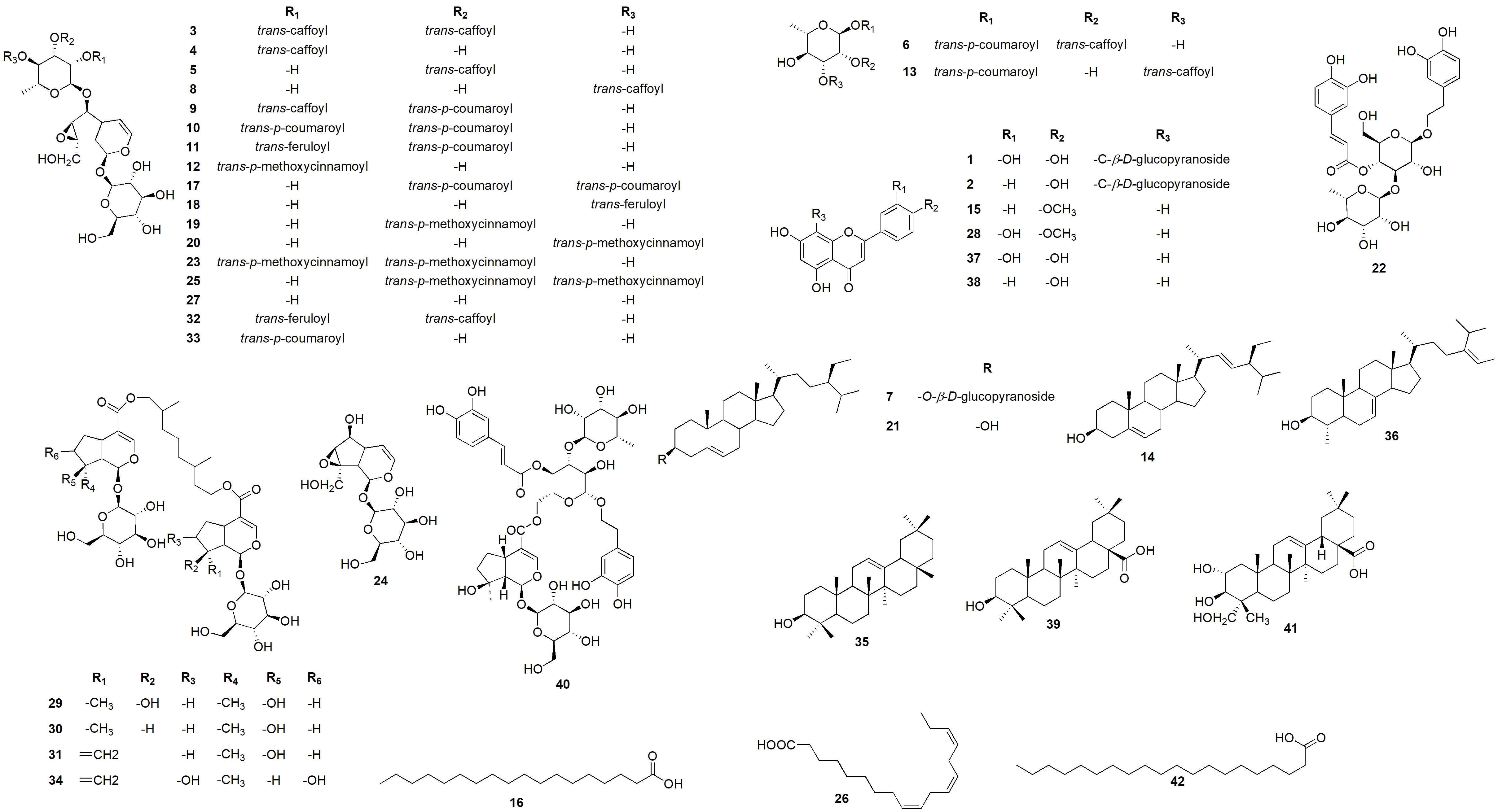

Supplement: Supplementary file 1 — Supplementary file1 (DOCX 226 KB) [file 11356_2022_20735_MOESM1_ESM.docx]
